# Factors affecting access to healthcare for young people in the informal sector in developing countries: a systematic review

**DOI:** 10.3389/fpubh.2023.1168577

**Published:** 2023-06-08

**Authors:** Ayomide Oluwaseyi Oladosu, Tual Sawn Khai, Muhammad Asaduzzaman

**Affiliations:** ^1^School of Graduate Studies, Lingnan University, Hong Kong, Hong Kong SAR, China; ^2^Research Affiliate, Refugee Law Initiative (RLI), School of Advanced Study, University of London, London, United Kingdom; ^3^Department of Community Medicine and Global Health, Institute of Health and Society, Faculty of Medicine, University of Oslo, Oslo, Norway

**Keywords:** access to healthcare, young people, informal sector, health, informal workers, developing countries

## Abstract

**Background:**

Young people are increasingly seeking employment in the informal sector due to increasing global unemployment. However, the precarious nature of work in the informal sectors, coupled with the high risk of occupational hazards, calls for a greater need for effective healthcare for informal sector workers, particularly young people. In addressing the health vulnerabilities of informal workers, systematic data on the determinants of health is a persistent challenge. Therefore, the objective of this systematic review was to identify and summarise the existing factors that affect access to healthcare among young people from the informal sector.

**Methods:**

We searched six data databases (PubMed, Web of Science, Scopus, ProQuest, Crossref, and Google Scholar), which was followed by hand searching. Then we screened the identified literature using review-specific inclusion/exclusion criteria, extracted data from the included studies and assessed study quality. Then we presented the results in narrative form, though meta-analysis was not possible due to heterogeneity in the study design.

**Results:**

After the screening, we retrieved 14 studies. The majority were cross-sectional surveys and were conducted in Asia (*n* = 9); four were conducted in Africa, and one in South America. Samples ranged in size from 120 to 2,726. The synthesised results demonstrate that problems of affordability, availability, accessibility, and acceptability of healthcare were barriers to young informal workers seeking healthcare. We found social networks and health insurance as facilitators of access for this group of people.

**Conclusion:**

To date, this is the most comprehensive review of the evidence on access to healthcare for young people in the informal sector. Our study finding highlights the key gaps in knowledge where future research could further illuminate the mechanisms through which social networks and the determinants of access to healthcare could influence the health and well-being of young people and thus inform policy development.

## Introduction

Globally, the informal sectors constitute over 60% of the workforce (1). While most developed countries have less proportion of informal workers, 93% of the global informal employment is in low- and middle-income countries, particularly in Asia and Africa (1). It is difficult to identify accurate representation of each nation on a country-to-country basis due to the flawed recording system in many developing countries. The apparent lack of records of their employment and, most often, lack of contract make it very difficult to reach them. However, on a demographic basis, young people account for one of the largest population groups in the informal sector (2). The International Labour Organization (ILO) describes the informal sector as a group of unincorporated production units owned by households, typically small and unregistered enterprises (3).

In fact, there is no universal definition of young people as it varies from context to context (4); however, the United Nations defines young people as people aged 15 to 24, and the African Union defines them as being between 15 and 35 years old (5). Approximately 77% of young workers are employed in the informal sector due to the rapidly changing dynamics of the labour market, such as increased unemployment (6). However, the jobs held by young people in the informal sector typically have low wages, long working hours, and no legal or social protections (6). These challenges, coupled with the daily occupational hazards, contribute greater need for healthcare among young people in the informal sector (7). As of yet, a limited amount of literature has investigated the factors that affect young informal workers’ access to healthcare, most of which has been conducted in Asia (7–10) and Africa (11–14). However, these studies focused on diverse groups of informal workers from different geographical contexts.

Access to healthcare has a profound effect on every aspect of an individual’s health, yet many young informal workers find it challenging to gain access, putting them at risk of poor health outcomes. Young people’s health challenges have demanded increasing attention on the global stage, as reflected in the Sustainable Development Goals (SDGs). One of the 17 sustainable goals is to “ensure healthy lives and promote well-being for all at all ages” (SDG 3) (15). To ensure this goal’s achievement (SDG 3), further progress needs to be made towards the achievement of universal healthcare (UHC). Achieving UHC requires the provision of healthcare to everyone, including those employed in the formal and informal sector. Unfortunately, people in the informal sector are mostly excluded from different forms of social protection including health insurance, as a result of their informality (7, 16).

Therefore, addressing this setback requires their access to healthcare. A recent study by Lee and Ruggiero (17) synthesised existing knowledge on the health and health equity implications of informal employment. However, a clear understanding of the factors that either positively or negatively affect their access to healthcare is needed for the health promotion of young informal workers to be achieved. In the light of the limited review-level evidence, our current systematic review aims to identify, analyse, and synthesise primary evidence on the factors affecting access to healthcare for informal workers, with the focus on young people. To the best of our knowledge, no study has systematically reviewed, analysed, and synthesised this evidence to point out gaps for future direction in the access to healthcare literature and policy implication.

## Methods

This systematic review explored the factors affecting access to healthcare and the health and well-being outcomes of young informal workers. In the larger review, health outcomes were grouped in a way that would offer great conceptual and practical value, for example, physical health, mental health, and health behaviours. However, this paper focuses only on the factors that affect access to healthcare for young informal workers.

Given that the purpose of this study was to synthesise existing empirical research to provide a consolidated overview of the evidence in this field and to put out gaps for future research, rather than the generation of a new theory, we adopted an integrative approach which enables the collation of different types of evidence (i.e., quantitative, qualitative, and mixed-methods) (18). The results from all the methods relevant to the identification and synthesis of data on the factors affecting access to healthcare (including factors hindering and facilitating access to healthcare).

### Criteria for inclusion

#### Types of studies

**Figure 1:**
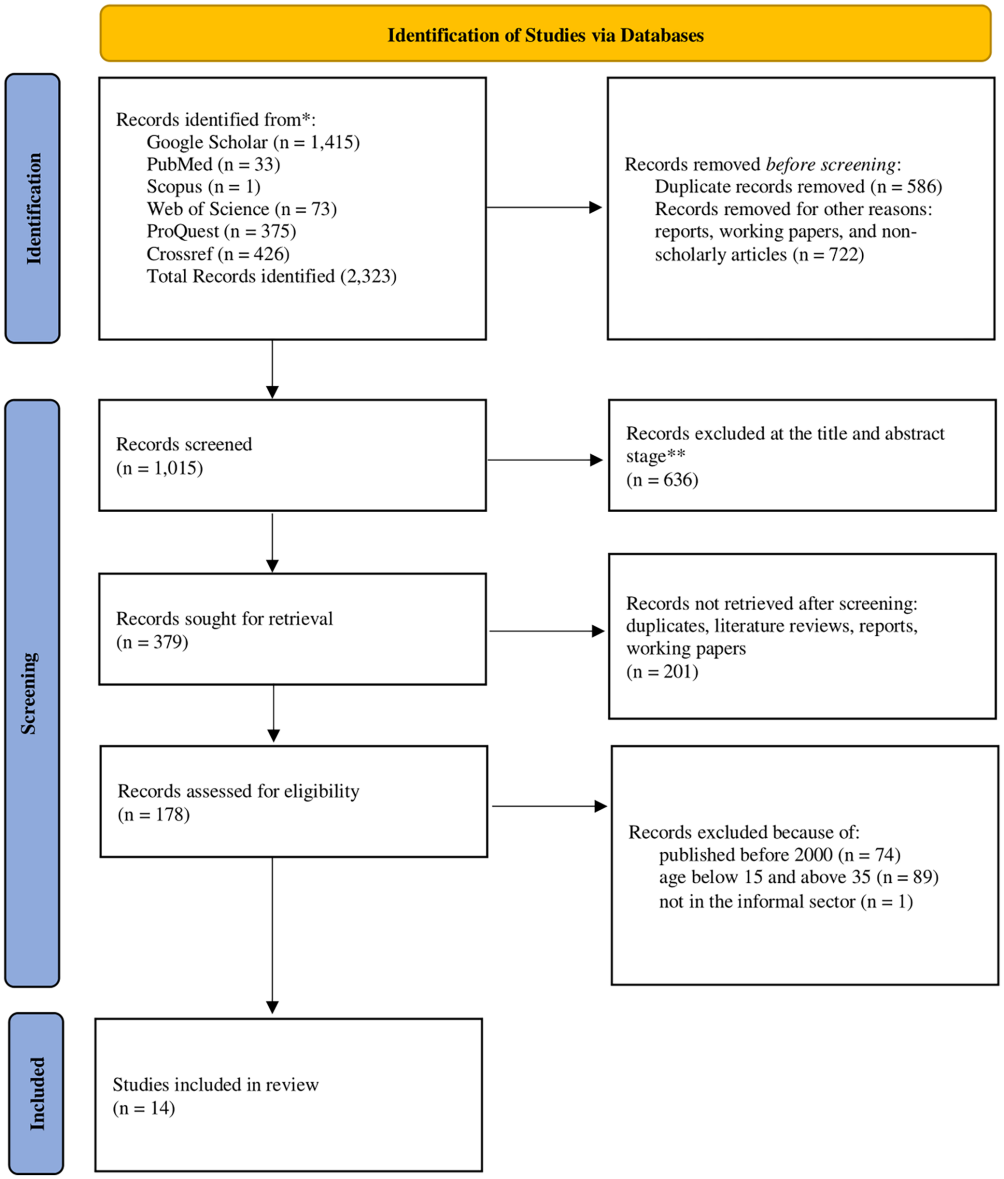
Search results and selection of papers (19).

Guided by the integrative approach, we sought to include empirical quantitative, qualitative, and mixed methods studies that were peer reviewed and published in the English language between the years 2000 and 2022. All articles dealing with either empirical or theoretical aspects of access to healthcare are included. After combining the results from the databases, duplicate articles were excluded. Publications without full texts, reports, working paper, magazines, letters to editors, correspondence, conference papers, and books were further excluded from the search results. Figure 1 summarises the search and selection process of the 14 articles in this review.

#### Types of participants

Studies were included if they focused on young people in the informal sector. The definition of young people in the literature varied in the way’s authors defined them. Therefore, we adopted a pragmatic approach, guided by the African Unions definition of young people, as people between the ages of 15 to 35 (5). This definition helped to collate large evidence from the different groups of people classified as young people in the literature. Studies where most of the participants were between 15 to 35 years were also included.

#### Dimension of access to healthcare

The study of access to healthcare for informal sector workers has been complicated by a lack of conceptual clarity around the term ‘access’, resulting in different access definitions (20). To ensure that all aspects of access to healthcare are included in this study this study adopted Peters et al.’s (21) access to healthcare approach to frame the discussion. They conceptualise access to healthcare services by using four dimensions—availability of service, affordability, geographical accessibility, and acceptability. This framework would help to capture the various factors affecting access to healthcare identified by the studies under this review.

### Databases and search strategy

#### Data sources

In this systematic review, six bibliographic databases were searched in September 2020 and updated in April 2022, including PubMed, Web of Science, Scopus, ProQuest, Crossref, and Google Scholar. We also hand-searched the reference list of retrieved articles and websites of organisations conducting research on the health and well-being of young people and informal workers. The websites included: The International Labour Organisation (ILO), the Rockefeller Foundation, and Women in Informal Employment: Globalizing and Organizing (WIEGO). Finally, hand searching was also conducted using Connected papers – an online software used to identify related and relevant literature—to ensure that all relevant papers were captured in the review.

#### Search terms and delimiters

As indicated earlier, the keywords included were broader than just access to healthcare. The keywords included: “young people,” “access to healthcare,” “access to health services,” “access to healthcare,” “artisans,” “informal workers,” “informal sector,” and “health,” in the title and abstract. We limited the searches to studies published within the year 2000 and 2022 and only studies published in the English language. Retrieved articles were stored in Mendeley. A study protocol was created, consisting of the study’s aim, key concepts, inclusion and exclusion criteria, and database search strategy. The review stages included retrieving search results from the approved databases, extracting duplicates, screening irrelevant data, assessing the retrieved studies, synthesising the data, and creating a report using a flowchart.

### Data collection and analysis

#### Selection of studies

The first step to selecting the studies included in this review was identifying and removing duplicates. All the articles identified from the search were first recorded in Microsoft Excel and tabulated under different headers such as author, title, year of publication, abstract, and cites. The results were initially stratified into six sheets based on the databases they were retrieved from. Each sheet contained the following contents: the article publication date, author(s), title, objective, methods, key findings, population, study site, strength, limitation, and conclusion. These contents helped simplify the data synthesis and cleaning. All the identified abstracts were subject to a two-stage screening process. The authors screened the title and abstracts independently, causing a long screening time, and articles that did not fit the inclusion criteria were automatically excluded. Where no abstract was available, the article was retained to the next stage, which involved screening the full-text subject to the inclusion and exclusion criteria. The entire screening process was double-checked by the two individuals who worked independently of each other.

#### Data extraction

A review-specific data extraction tool was designed to enable the extraction of data from all studies with different designs. The key elements of the data extraction included the context of the study, such as the geographical location and year of the study, the aims and objectives of the studies, the study design, the participants—the age and the type of informal work—the main findings, and the strengths and limitations. This was also conducted with a review by two individuals.

#### Quality appraisal of selected publications

The quality appraisal was conducted at the same time as the data extraction. The articles selected for this review were qualitatively evaluated and further reviewed by the first author’s two supervisors, who served as external reviewers to ensure that both the selection process and the articles selected were of premium quality. The quality of the articles was assessed before the final selection for review. The assessment was done based on the five-criteria framework designed by Dixon-Woods et al. (18). The five criteria include: were the aims and objectives clearly stated in the article; was the design specified, and was it suited for the research goal; was the research process clearly explained; was there sufficient data to support the interpretation and conclusion of the study?; and was the analytical method clearly explained and appropriate for the study. A tick symbol (✓) was used to show that an article met a criterion, while a negative symbol (X) indicated that the article did not meet a criterion. Each tick symbol represented one point, and each negative symbol represented 0, for a total of five quality assessment points for each article. Scores of 2 and below were classified as low, 3 was moderate, and 4 and above were high (see Table 1 for quality assessment).

**Table 1:** Quality assessment of studies included.

S/N	Criteria	Akazili et al. (11)	Sychareun et al. (7)	Webber et al. (8)	Salman et al. (26)	Natha Mote (22)	Namsomboon and Kusakabe (10)	Nam et al. (9)	Gichuna et al. (13)	Dartanto et al. (25)	Boateng et al. (12)	Ahmed et al. (23)	Dartanto et al. (24)	Barasa et al. (14)	Ganem dos Santos et al. (27)
1.	Are the aims and objectives clearly stated in the publication?	✓	✓	✓	✓	✓	✓	✓	✓	✓	✓	✓	✓	✓	✓
2.	Is the research design specified, and is it suitable for the research goal?	✓	X	✓	✓	✓	✓	✓	✓	✓	✓	✓	✓	✓	✓
3.	Is the research process clearly explained?	✓	✓	✓	✓	X	✓	✓	✓	X	✓	✓	✓	X	✓
4.	Is there sufficient data to support the interpretation and conclusion of the study?	✓	✓	✓	✓	X	✓	✓	✓	✓	X	✓	✓	✓	✓
5.	Is the analysis method clearly explained and appropriate for the study?	✓	X	✓	✓	X	✓	X	✓	✓	✓	✓	✓	X	✓
6.	Total score	5	3	5	5	2	5	4	5	4	4	5	5	3	5

### Data analysis and synthesis

The results are presented using a narrative approach because of the small number of studies represented in this study. The results were first summarised and then synthesised and adapted the approach originally described by Ramirez et al. (28). Specifically, the results were grouped into two categories of factors affecting access to healthcare: results that showed factors associated with barriers and facilitators of access to healthcare. Many of the studies in this review reported on various factors that hindered young informal workers from accessing healthcare (barriers to healthcare access). A few other studies reported on the factors that facilitated healthcare access. The heterogeneity in the outcomes and study designs of the included studies prohibited meta-analysis. Therefore, the result was presented in a narrative format.

## Results

### Study selection

The study selection process followed the PRISMA (Preferred Reporting Items for Systematic Reviews and Meta-analysis) guidelines (19). The search and screening stages are represented in Figure 1. After removing duplicates, the search yielded 1,015 unique studies screened using the inclusion/exclusion criteria. The majority (*n* = 636) of the studies were excluded at the title and abstract levels. A second-level screening was conducted, which identified and excluded 201 records, including unidentified duplicates, literature reviews, reports, and working papers. A further 161 records were excluded because they were published before the year 2000 (*n* = 74) and did not fit our definition of young people [including the one article that does not represent young informal workers (*n* = 90)]. Finally, a total of 14 studies were included in the total sample that are reported here.

### Description of the included studies

The quality appraisal ratings and key descriptive information for each of the 14 studies included is presented in Table 1. Thirteen studies were rated high quality, two were rated moderate quality, and 1 study was assessed as being low quality (See Table 1). Following the data extraction and synthesis, the barrier factors to healthcare access were grouped into four coherent categories based on the four dimensions of access to healthcare (affordability, availability, accessibility, and acceptability); the facilitating factors were grouped into three categories (social networks, health insurance, and technology).

In terms of the study design adopted by the articles in this review, 53.3% (*n* = 8) employed a quantitative research design, 33.3% (*n* = 5) were qualitative, and 13.3% (*n* = 2) utilised a mixed-method approach (See Table 2). The majority (*n* = 10) of quantitative studies (including mixed methods) employed a cross-sectional and survey data collection method; the sample also included 1 quasi-experimental study. Among the qualitative studies, the three studies (7, 13, 14) employed solely in-depth interviews (IDIs), 1 study (8) employed solely focused group discussions (FGDs), and 3 studies employed both IDIs and FGDs (9–11). The majority (*n* = 14) of the studies were conducted in developing countries. This did not come as a surprise, given the report that shows that 93% of the world’s informal employment is located in developing and emerging economies (29). In terms of geographical locations, the majority (*n* = 9) of the studies were conducted in Asian countries (Bangladesh, Cambodia, India, Indonesia, Laos, Nepal, Pakistan, Thailand, and Vietnam). This was followed by studies in Africa (*n* = 4; Ghana and Kenya). Study in South America (*n* = 1; Brazil) was the least represented (see Table 2). In terms of urban/rural settings, 57% (*n* = 8) of the studies were carried out in urban communities, 14% (*n* = 2) of the comprehensive studies were conducted in rural communities, and 29% (*n* = 4) conducted in both urban and rural communities (see Table 2).

**Table 2:** Description of studies included.

S/N	Author(s)	Year	Objective	Design	Sample size	Age (years)	Study Site	Types of informal workers
1.	Webber, G., Spitzer, D., Somrongthong, R. et al.	2012	To assess access to sexual and reproductive health services for migrant women who work as beer promoters.	FGD and Survey (Mixed-Method)	390	Mean age = 24.2	Cambodia, Laos, Thailand and Vietnam	Female beer promoters
2.	Salman et al.	2015	To assess the prevalence of chronic diseases and workplace physical trauma among low-income workers.	Cross-sectional Survey (Quantitative)	707	15–35	Karachi, Pakistan	Varied
3.	Akazili, J., Chatio, S., Ataguba, J. E. O. et al.	2016	To explore the factors affecting informal workers access to health care services in Northern Ghana	Focus Group Discussions (FGD) and in-depth interviews (Qualitative)	21	18 and above	Rural Eastern and Western Districts of Northern Ghana.	Hairdressers, head potters, farmers and bar attendants.
4.	Teguh et al.	2016	To examine why informal workers are reluctant to join the national health insurance even though the programme’s benefits are very generous.	Survey (Quantitative)	400		Indonesia	Varied
5.	Natha Mote	2016	To examine the health status, occupational and environmental health hazards among ragpickers working in the dumping ground of Shivaji-Nagar and Govandi Slums of Mumbai, India.	Survey (Quantitative)	120	18 and above	Mumbai, India	Ragpickers
6.	Sychareun et al.	2016	To examine the interplay between the experience of informal work and access to healthcare.	In-depth interviews (Qualitative)	24	18 and above	Vientiane city, Laos People Democratic Republic	Female beer promoters.
7.	Barasa et al.	2017	This paper analyses the perceptions and experiences of informal sector individuals in Kenya concerning enrolment with the NHIF.	In-depth interview (Qualitative)	39	Not stated	Kenya	Varied
8.	Boateng, S., Amoako, P., Poku, A. A. et al.	2017	To analyse the factors associated with enrolment in and renewal and utilisation of the NHIS among migrant female head porters in the Kumasi Metropolis.	Survey (Quantitative)	392	15–35	Kumasi, Ghana	Head potters
9.	Ahmed et al.	2018	To estimate the impact of a Community-Based Health Insurance (CBHI) scheme on healthcare utilisation from medically trained providers (MTP) by informal workers.	Quasi-experimental (Quantitative)	1,292	Not stated	Bangladesh	Varied
10.	Namsomboon and Kusakabe	2011	To examine women homeworkers’ access to healthcare services in Thailand.	Mixed-methods (Survey, IDI, and FGDs)	415	15–35 (75%)	Thailand	Female homeworkers
11.	Dartanto, T., Halimatussadiah, A., Rezki, J. F. et al.	2020	This study aimed at exploring the critical factors that affect the compliance behaviour of informal sector workers (PBPU members) in regularly paying their insurance premiums.	Survey (Quantitative)	1,210	Not stated	Indonesia	Varied
12.	Fabiana Sherine Ganem dos Santos et al.	2020	To determine the prevalence of syphilis and other sexually transmitted infections (STI’s) among waste pickers who worked at the open dumpsite.	Survey (Quantitative)	1,025	18 and above	Brazil	Waste pickers
13.	Gichuna et al.	2020	To highlight specific effects of COVID-19 and related restrictions on healthcare access for sex workers in informal settlements in Nairobi, Kenya.	In-depth interview (Qualitative)	117	16–33 (83%)	Nairobi, Kenya	Sex workers
14.	Tien Nam et al.	2020	This study aimed to understand the factors affecting access to health services among waste collectors in Hanoi, Vietnam.	In-depth interview and FGD (Qualitative)	49	Not stated	Vietnam	Waste collectors

Ten informal working populations were represented in this review; they include female beer promoters, bartenders/attendants (Cambodia, Laos, Thailand, and Vietnam), head potters, farmers, and hairdressers (Ghana), female homeworkers (Thailand), rickshaw pullers, ragpickers, waste collectors (Brazil and India), sex workers (Kenya). Three studies (7, 10, 13) had female-only respondents.

The 14 studies under review had a considerable variation in sample size; the qualitative studies ranged from 21 to 49 respondents for the focused group discussions and 12 to 117 respondents for the in-depth interviews. In contrast, the quantitative studies had larger sample sizes ranging from 120 to 2,726. One quantitative study (22) did not have an adequate sample to justify its result (see Table 2).

### Factors affecting access to healthcare among young informal workers

#### Barriers to access to healthcare

Several factors were shown to hinder access to healthcare services for informal workers across different study locations. For the sake of this study, all the elements would be grouped under four dimensions of healthcare access (affordability, availability, accessibility, and acceptability) following the conceptual framework of access to healthcare (21).

##### Affordability of healthcare

Eight studies (8–13, 24) discovered affordability as one of the significant barriers to healthcare for young informal workers across diverse industries. Five studies (7–11) identified the cost of seeking formal healthcare as one of the problems young informal workers are unable to seek the healthcare they need. Sychareun et al. (7) and Akazili et al. (11) argue that informal workers are lower earners and, therefore, they may undergo financial distress in accessing healthcare. Two studies (8, 9) identified the direct cost of transportation as another component of affordability that hindered young informal workers from accessing healthcare because of the distance from where they live to the hospitals. Five studies (8, 9, 11, 12, 24) identified the absence of health insurance for young informal workers, leading to high out-of-pocket payments and high costs of healthcare. There was also evidence that other indirect affordability factors, such as long waiting time (9) and the loss of daily/weekly wages and jobs (7, 11), hindered young informal workers from seeking healthcare. Absence from work as a result of going to the hospitals may result in pay cuts or loss of jobs for these groups of people due to the long waiting time many experiences at the health centres (7).

##### Accessibility of healthcare centres

Five studies (8–11, 30) identified problems of accessibility as some of the barriers to healthcare among young informal workers. Two studies (8, 9) found that the distance to hospitals and travel time were limitations to how much informal workers could seek healthcare. As a result of the distance and travel time, particularly for those who reside in rural areas (30), many informal works result in obtaining services from informal drugstores closer to them (9). Two studies (8, 11) observed that the means of transportation was another factor. Because many informal workers lived outside of town, it was more challenging to frequently visit the hospitals or health centres due to bad roads (11) or less frequent public transport systems (8). As a result of low educational qualifications, many young informal workers lacked the knowledge or information related to navigating health systems (10). Their educational qualification also made it difficult to understand the complex billing system in many hospitals; as a result, many of them self-medicated if they had no one to support them (9).

##### Acceptability of healthcare

Four studies (7, 9–11). Three components of service acceptability were identified in the above four studies. The first one is the perceived poor treatment from health personnel. The formal healthcare centres were often seen as a place of last resort after self-medication and other sources of treatment proved futile (7). This was simply because these informal workers perceived that the healthcare officials would not warmly receive them because of their socio-economic status, particularly their level of income and education (7–9). The second component is the poor quality of healthcare informal workers receive. Two studies (7, 10) found that informal workers in Laos and Thailand perceived the quality of healthcare and the drugs they received as poor. This was associated with their report that the healthcare systems around them were not sufficiently developed (7). The third component this study identified is the perceived cleanliness of the health centres. One study (8) found that informal workers refused to go to the health centres because of the perceived state of sanitation.

##### Availability of healthcare resources

Availability of service is measured as the opportunity to access quality healthcare service as and when needed (21). The concept of healthcare service availability includes healthcare workers’ availability, medication stock, long waiting times, and equipment. Tien Nam et al. identified these five components of healthcare availability (infrastructure, human resources, medical equipment, and medicine) as barriers to healthcare service among waste collectors in Hanoi, Vietnam (9). Seven studies (7–9, 11, 13, 24) reported availability as one of the service barriers. The problems of availability were linked to the lack of available medical equipment (13), insufficient healthcare personnel, particularly doctors (9, 11, 13), and inconvenient opening and closing hours (13). Gichuna et al. found that female sex workers were denied access to sexual reproductive healthcare because there was no specialised equipment to provide the service they needed (13). However, this study was conducted at the peak of the Covid-19 pandemic, when the movement of goods and services was restricted (13). In Ghana and Vietnam, most informal workers live in the rural areas or the outskirt of towns, where there are no sufficient doctors or nurses to cater for the healthcare needs of the multitude of informal workers in those areas (9, 11). Finally, the opening and closing hours of the health centres were unsuitable for these young informal workers because many of them resumed very early to work or closed late at night after most hospitals or clinics had closed (7, 9, 11, 13). Absence during working hours puts them at risk of pay cuts or loss of jobs (7). In the case of female sex workers in Kenya, Covid-19 was the reason for the disruption in the opening and closing hours of the health centres (13). In sum, the availability of healthcare resources is a significant barrier to healthcare for young informal workers, majorly, supply side factors, such as the availability of doctors, equipment, medicines, and even the opening and closing hours of health centres are barriers to seeking healthcare for many young people in the informal sector.

#### Facilitators of access to healthcare

Two factors (social network and health insurance) that facilitate access to healthcare for young informal workers were identified from seven studies (7–9, 11, 12, 24, 31). Five studies (7–9, 11) found that social networks, such as friends, families and co-workers, facilitated entry into healthcare systems by lending or gifting cash to their peers, siblings, children or relatives who fell under this category. Not just money but social networks were instrumental in choosing the healthcare institutions they sought (7, 8, 11). Finally, Webber et al. (8), Nam et al. (9), Akazili et al. (11), and Teguh et al. (24) identified that being enrolled in the government’s health insurance scheme was significant in overcoming the financial barrier of seeking healthcare.

## Discussion

### Summary of results

The primary aim of this systematic review was to identify, analyse and synthesise empirical evidence on the factors affecting the access to healthcare of young people in the informal sector. In doing so we assessed 14 studies, to the best of the author’s knowledge, making it the first and the most comprehensive systematic review to focus specifically on the access to healthcare for young people in the informal sector in developing countries. The relatively large body of evidence included in this review supports the conclusion that young people in the informal sector face significant barriers (affordability, availability of healthcare resources, accessibility of healthcare systems, and the acceptability of healthcare services) to healthcare. However, some factors, such as social networks and health insurance, if prioritised by policymakers in majorly developing countries, can improve young informal workers’ access to healthcare.

### Barriers to access to healthcare

This study identified several factors hindering healthcare access among young informal workers in developing countries. Each of these factors are grouped into the four dimensions of access to healthcare (affordability, availability, accessibility, and acceptability) utilising Peters et al. (21) access to healthcare framework. Although young informal workers are affected by each of these factors, we discussed based on the major hindering factors., that were identified as being responsible for young informal workers’ failure to get health care.

#### Affordability of healthcare services

Several factors were linked to the problem of affordability. The most common affordability-related problem affecting access to healthcare for young informal workers was the cost of treatment (7–11). Healthcare financing for informal workers is mainly out-of-pocket; this is primarily because informal workers are largely excluded from social protection schemes such as health insurance. However, the situation is more challenging for young people than for older people. Unlike the older population, young people are hardly in trade unions or associations that can provide financial support for their healthcare needs. In addition, having and not having health insurance has been identified as a barrier or a pathway to overcoming financial barriers to healthcare services. In terms of promoting access to healthcare services, Tien Nam et al. identified the presence of health insurance as a potential facilitator of access to healthcare among informal waste collectors in Hanoi, Vietnam (9). A study by Ahmed et al. using healthcare utilisation as their variable for access to healthcare (23). The study found that healthcare utilisation among informal workers was significantly higher in the insured group (50.7%) than in the uninsured group (39.4%) (23, 32). Their regression analysis further reported that the regression analysis demonstrated that the community-based health insurance (CBHI) beneficiaries were two times more likely to utilise healthcare. While Ahmed, et al., Boateng et al., and Teguh et al., similarly identified that health insurance schemes were necessary facilitators for healthcare access in Ghana (12, 23, 24, 25). However, except in a few developing countries where health insurance is offered and subsidised for informal workers, many young informal workers are unable to obtain health insurance.

The second factor is linked to the income of young informal workers. One may argue that informal workers generally have low incomes. However, the problem of income is worse for young people than for the general adult population (33). As new entrants into the labour market with little or no skills and experience, young people are usually at the lowest level of the income scale (33). Their earning is usually not enough to save and cover healthcare needs. This makes it more difficult for young people to access healthcare than other age groups of informal workers. Another factor is the cost of being away from work as a result of seeking healthcare (7, 8, 11). Absence from work is extremely costly for young informal workers since they lose their income for the duration of their absence and can even result in the loss of their employment if a prolonged medical treatment period is required, in addition to transportation expenses. Further, most informal employment sectors have no legal or formal social benefit arrangements, such as sick leave pays benefits when workers become ill (3). Therefore, absence from work to seek healthcare almost equates to a loss of income or job. This problem is common among young people because, unlike older people, they are more likely to be apprentices or employees with no autonomy (33, 34).

The last problem identified from the studies under review is that of transportation cost (7, 8, 11). The problem of affordability, either directly or indirectly, has been linked to the poor wages of young people in the informal sector (7–9, 11). This is evident in the case of young female beer promoters in Laos (7), head potters and hairdressers in Ghana (11) and female bartenders in Laos, Thailand, and Vietnam (8). This study’s finding is similar to previous research, which found that disadvantaged populations had less access to healthcare services due to poverty or lack of financial capacity (21, 35). Most informal workers do not earn enough money to save or care for their health needs. As a result, it is not surprising that most of the studies in this review revealed that most young informal workers could not afford health care because they likely do not have much to cover extra expenses beyond their basic needs.

To overcome the affordability barrier, young informal workers relied on their social networks, such as friends, family members (parents, older siblings and relatives), neighbours, co-workers, customers and employers, to access healthcare (7–9, 11). Social networks provided instrumental support to young informal workers through lending or gifting cash to seek healthcare. Unlike a previous study (36) on social networks, and access that found that weak ties, such as members of associations and distant relationships, were more important for accessing resources, this study found that in the case of young informal workers, close social ties were the most important social relationships for accessing healthcare. For instance, Sychareun et al. and Webber et al. found that parents and peer networks were instrumental for young informal workers to overcome financial barriers to healthcare access (7, 8). Only one study (11) found that distant relationships, such as employers, were significant in providing financial resources to young informal workers who access healthcare. However, none of these studies measured how social networks can promote access to healthcare for young informal workers. The generalisation of social networks’ influence on access to healthcare may be difficult based on contextual factors like ethnicity, religion, culture, and economic activities that can determine the extent of a social network–health relationship (37).

#### Availability of healthcare resources

According to the findings, the second most significant dimension of healthcare for young people is the availability of healthcare. Although the availability of healthcare (shortage of healthcare resources such as human resources, equipment, and medicines) is a dominant healthcare problem in many developing countries, the problem is particularly significant for informal workers, particularly women (7, 8, 11). For example, young female bartenders and beer promoters in Thailand, Vietnam, and Laos DPR are hesitant to seek healthcare due to feelings of shame and fear following harassment and stigmatisation from their customers (7, 8). However, there were no nearby healthcare services tailored to young people’s needs in such situations. This lack of available healthcare may result in even greater levels of fear, anxiety or depression, and even substances use as a coping strategy among young female informal workers.

A further problem for many developing countries is that healthcare personnel and equipment are very limited, leading to long wait times for treatments in many public hospitals (21). Those who cannot afford private medical care find this aspect especially challenging. This is particularly a concern for young informal workers because, as mentioned previously, absence from work often equates to a loss of income and sometimes loss of jobs. This can cause a considerable financial burden on families and lead to further poverty among young informal workers in developing countries. Furthermore, COVID-19 also exacerbated healthcare availability problems for young informal workers (13). The supply of other healthcare resources likely declined due to the high priority placed on COVID-19 resources. This led to fewer healthcare services for non-COVID-19-related issues (38, 39). In some cases, this has decreased the quality of care for those seeking treatment for other medical issues (13, 39). Furthermore, appointment wait times may have increased, causing further delays in treatment.

#### Acceptability of healthcare

The third dimension of access to healthcare that significantly affected the access to healthcare for young informal workers was acceptability. Based on the evidence from the studies in this review, the problem of acceptability can be viewed from two sides. On the one hand, the problem of acceptability was a supply-side problem; on the other, it was a demand-side problem. On the supply side, the attitude of healthcare workers and stigmatisation served as barriers to access healthcare for young informal workers (7–9, 11, 13, 14). First, young informal workers reported negative attitudes, such as neglect and insults from healthcare operatives towards them because of their socio-economic status. Furthermore, young female informal workers reported incidences of stigmatisation because of their jobs as bartenders or beer promoters (7, 8). They were perceived as sex workers and treated poorly (7, 8). These factors lead to demand-side factors such as fear and lack of trust and confidence in healthcare providers, which hinder many young people from seeking healthcare even when they can afford it (30). This can lead to delayed diagnosis and treatment of health conditions, resulting in poorer health outcomes. Additionally, young women may also experience social isolation due to their perceived status as sex workers, which can further compound their health and safety risks. Evidence from studies in low-and-middle-income countries suggests a prevalence of poor-quality healthcare, which is attributed to between 5.7 and 8.4 million deaths annually (32). However, the extent to which it affects the health of young informal workers was not stated in any of the studies included in this review. To overcome these challenges, young informal workers relied on peer networks to choose where to seek healthcare (7, 8). Peer networks were important in the healthcare-seeking behaviour of young informal workers. The choice of whether to seek care was not only based on the direct previous experience of young informal workers but also on the experience of their friends or colleagues. Studies have shown that young people rely on recommendations from friends for where to seek care because of confidentiality issues (30). To this end, peer networks can act as a support system that encourages young informal workers to seek out the healthcare they need. Additionally, these networks can provide information on the best place to seek care and ensure that young informal workers receive the treatment they need in a safe and secure environment.

#### Accessibility of health centres

Finally, the last major factor impeding young informal workers’ access to healthcare was accessibility. Travel distance, time, and inadequate transportation (7, 8, 11) were the two biggest hurdles to geographical accessibility. While this is a concern for all informal workers in most developing countries, it is exacerbated for young informal workers due to the time away from work, which may result in income loss. This was especially noticeable among young informal workers who do not reside or work in urban areas (11), with sparse healthcare services and inadequate transportation infrastructure. According to Amoah and Peters et al., many developing nations encounter issues with the problems accessibility of healthcare services due to poor transportation infrastructure (21, 40).

Furthermore, COVID-19-induced inaccessibility was identified due to physical distancing measures implemented by governments to reduce the spread of the Coronavirus. Individuals could not travel out of town to obtain healthcare (41, 42). This made it difficult for many young informal workers in developing countries to receive the care they needed. To address this, telehealth services were introduced in many advanced countries to provide healthcare services remotely (43). However, for those in developing countries with limited capacity, especially for underprivileged populations like young informal workers, accessing healthcare remained a significant challenge. For example, female sex workers in Kenya face a significant challenge in travelling to the hospital to obtain sexual and reproductive healthcare due to the social distancing measures in Kenya (13).

### Strength and limitation

To the best of the author’s knowledge, this review is the first to systematically collate, analyse and synthesise empirical peer-reviewed evidence of different research designs on access to healthcare among young people in the informal sector. This study brings together factors affecting access to healthcare for young informal workers across different developing countries. Also, the included studies were rated from moderate to high, which strengthens the quality that can be drawn from the synthesised result. However, this study has some limitations. Our study also has difficulty capturing all groups’ views, although access to healthcare remains a significant focus area for researchers and policymakers globally. Therefore, it is essential to note some of the limitations of this study. Most of the studies reviewed in this article are not nationally representative and pertain to a small population segment. Therefore, caution should be taken when interpreting the results of this study. There is a need for further research to understand whether these findings will be applicable to a wider population. We acknowledge that all relevant literature may not have been captured, as journals are known to favour papers based on statistical significance; such papers may not have been represented here. Although the systematic review was thoroughly conducted and efforts were made to ensure the search strategy was as robust as possible, the success in capturing studies was also dependent on adequate indexing of studies within the databases. Finally, non-journal papers, unpublished works, and thesis were not included in this review. Such publications may shed light on the issues influencing young informal workers’ access to healthcare and good health.

### Policy implication

Through this systematic review, we have tried to investigate the major factors affecting young informal workers’ health and well-being. We have also discussed how and to what extent such factors like social networks (social relationships) and health insurance impacted their life. The ultimate goal of researching access to healthcare is to provide policy recommendations for improving the population’s health and well-being (44). Our findings suggest that governments in developing countries should not neglect the health needs of young informal workers as a significant stakeholder and contributor in the national economy. To achieve universal healthcare and to ensure healthy lives and well-being for all (SDG-3), governments in developing countries need to collaborate with NGOs and researchers in delivering healthcare services to young informal workers.

### Future research direction

In some developing countries where national health insurance schemes do not protect informal workers, assessing this variable for them may be unrealistic. Therefore, future studies need to be moved beyond identifying the factors affecting healthcare access. Assessing the extent to which these factors help improve young informal workers’ health is rather crucial. The findings of such investigations may benefit governments, researchers, policymakers and non-governmental organisations interested in improving young people’s health, particularly those in the informal sector.

## Conclusion

Our study findings suggest that factors connected with the affordability, availability, accessibility and acceptability of healthcare represent some of the barriers many young informal workers encounter in seeking healthcare. Affordability represented the most significant barrier to healthcare that young informal workers face due to increased out-of-pocket healthcare expenditure and low wages of young informal workers in developing countries. This finding suggests that subsidised health insurance should be extended to young informal workers in developing countries. Such a policy, however, should be tailored to the needs of each country. Developing countries need additional investment to meet young informal workers’ healthcare needs. Young people are the lifeblood of any nation, so an investment in their health is an investment in its national growth and development. Moreover, the findings suggest that informal sectors should be more regulated in developing countries to enhance labour rights standards and minimise the exploitation of young informal employees.

Further, our findings indicate that social networks and health insurance contribute to improved health outcomes. While previous studies provide evidence on the factors that affect access to healthcare for only a few groups of informal workers, it remains unclear if these factors can be generalised across all young people in the informal sector. Also, the extent to which these factors affect the health of young informal workers remains unknown. The findings from this review point to significant gaps in access to healthcare literature and suggest areas of future research directions.

## Author contributions

AOO was responsible for the concept development and initial manuscript drafting. TK was responsible for the search. AOO, TK, and MA were responsible for the data analysis and synthesis. MA reviewed the manuscript. All authors contributed to the article and approved the submitted version.

## Conflict of interest

The authors declare that the research was conducted in the absence of any commercial or financial relationships that could be construed as a potential conflict of interest.

## Publisher’s note

All claims expressed in this article are solely those of the authors and do not necessarily represent those of their affiliated organizations, or those of the publisher, the editors and the reviewers. Any product that may be evaluated in this article, or claim that may be made by its manufacturer, is not guaranteed or endorsed by the publisher.

## Abbreviations

CBHI: Community-Based Health Insurance
FGD: Focused Group Discussion
IDI: In-depth interview
ILO: International Labour Organization
NHIS: National Health Insurance Scheme
UHC: Universal Healthcare
SDG: Sustainable Development Goals (SDGs)
SSA: Sub-Saharan Africa
